# An Anesthesiologist's Moral Distress With Organ Procurement: A Narrative Review

**DOI:** 10.7759/cureus.88765

**Published:** 2025-07-25

**Authors:** Prishay Johri, Rohan Rani, Christopher Spevak, Claudia Sotomayor, Nicole Cornish, Lorenzo De Marchi, Harold Wain, Allen Roberts

**Affiliations:** 1 Anesthesiology, Georgetown University School of Medicine, Washington, USA; 2 Bioethics, Georgetown University Medical Center, Washington, USA; 3 Anesthesiology, Georgetown University Medical Center, Washington, USA; 4 Psychiatry, Uniformed Services University of the Health Sciences, Washington, USA

**Keywords:** anesthesiology anesthesia, anesthetist ethics, bioethics, brain death, circulatory death, dead donor rule, ethical dilemma, moral distress, normothermic regional perfusion (nrp), organ procurement

## Abstract

Organ procurement procedures have evolved significantly over the past decade, resulting in new moral uncertainties. The dead donor rule (DDR), a foundational principle, mandates that organ procurement occurs only after death is confirmed. However, developments such as donation after circulatory death (DCD) and normothermic regional perfusion (NRP) have raised ethical issues, creating moral distress and moral injury among anesthesiologists. This review highlights the recent changes in the organ procurement processes and the potential impact on anesthesiologists and also discusses the strategies to prevent and manage moral distress. A narrative review was conducted using a structured search strategy across Ovid MEDLINE, Embase, and Web of Science. The search included the terms "anesthesiology", "moral distress", "dead donor rule", "brain death", and "normothermic regional perfusion". Of the 77 identified articles, 18 met inclusion criteria focusing on the ethical, psychological, and educational aspects of the anesthesiologist’s role in organ procurement. The increasing use of novel organ procurement techniques such as NRP after DCD may result in moral distress among anesthesiologists. While the literature on moral distress continues to expand in the healthcare field, more needs to be written on this topic with anesthesiologists involved in organ procurement. Anesthesiologists are participating in novel organ procurement techniques with minimal control of decision-making. As a result, moral distress may occur with negative consequences. Solutions to the identification and prevention of moral distress with organ procurement include expanded ethics education, peer and mentor support, and institutional support. Addressing these issues can empower anesthesiologists to navigate complex scenarios, mitigating moral distress and increasing wellness and patient safety.

## Introduction and background

In recent years, moral distress has garnered increasing attention across various disciplines. Initially explored within the nursing field, it has since been broadened to encompass the experiences of all healthcare professionals. This expansion reflects a growing recognition of medical personnel's ethical challenges in diverse clinical settings. There must be an ethical component inherent in the situation to constitute moral distress. In other words, moral distress arises when individuals are confronted with a situation in which they cannot act according to their moral standards due to external constraints, thereby involving a conflict between ethical values and professional or situational limitations.

The field of organ donation and transplantation is no exception to experiencing moral distress, mainly because it has undergone substantial advancements in recent years, leading to the emergence of new challenges that carry inherent ethical implications. These ethical dilemmas may evoke discomfort among healthcare providers, complicating the decision-making process and care delivery.

One significant issue with profound ethical implications is the determination of death. Two primary definitions of death have traditionally guided organ donation protocols: brain death and circulatory death. Brain death refers to the irreversible cessation of all brain activities, including the brainstem, while circulatory death is defined as the irreversible cessation of circulatory and respiratory functions. These criteria are supported by the Uniform Determination of Death Act (UDDA), which provides a legal framework for death determination in the United States [1]. The UDDA is a model statute created for adoption by state legislatures, which codified that death may be established in an “individual who has sustained either (1) irreversible cessation of circulatory and respiratory functions or (2) irreversible cessation of all functions of the entire brain, including the brain stem. A determination of death must be made in accordance with accepted medical standards” [2].

A fundamental ethical principle in organ donation is the dead donor rule (DDR), which mandates that organ procurement should only occur after a donor has been declared dead and that the procurement process itself must not cause death. This rule has historically served as a safeguard to maintain public trust and establish clear ethical boundaries in organ donation. However, recent advancements in organ procurement techniques, particularly normothermic regional perfusion (NRP), have introduced complexities that challenge these boundaries and raise questions about the framework underpinning organ donation practices [1,3].

NRP is a technique designed to restore circulation to selected organs after death has been declared while preventing perfusion to the brain. This approach, aimed at improving organ viability, includes "A-NRP" for abdominal organs and "TA-NRP" for thoracoabdominal organs. In "A-NRP," the descending aorta is either clamped or obstructed with a balloon, and organ perfusion is maintained via a heart and lung machine. This technique, therefore, does not require carotid clamping. While NRP has demonstrated potential for better transplant outcomes, it presents ethical dilemmas for anesthesiologists and other healthcare providers. Truog et al. described NRP as the "next frontier" in resuscitative practices. Still, he acknowledged its challenges by partially restoring postmortem circulation, effectively challenging the finality of circulatory death and the DDR [3,4].

Professional organizations have presented differing opinions on NRP. In 2021, the American College of Physicians raised concerns about the potential of NRP to undermine public confidence in organ donation, warning that selective restoration of circulation could cloud the ethical line between life and death [5]. However, the American Society of Transplantation has expressed support for NRP, highlighting its potential to increase organ viability and improve transplant outcomes while emphasizing the importance of adhering to ethical principles and maintaining transparency in its application [6].

The legal framework surrounding NRP adds complexity to its implementation. Glazier and Capron highlighted that the practice of NRP raises potential conflicts with the Uniform Determination of Death Act (UDDA), which serves as the legal standard for determining death. The UDDA defines death based on the irreversible cessation of circulatory or brain functions. NRP, by restoring partial circulation post-death declaration, introduces complexities that may challenge the interpretation of these criteria. While the UDDA provides a uniform framework, its application to NRP varies, creating ethical and legal uncertainties that complicate anesthesiologists' roles in ensuring compliance with professional standards and the DDR [1].

Given the increasing prevalence of NRP in organ donation, anesthesiologists face unique challenges. This narrative review explores the moral distress and moral injury caused by these ambiguities. It analyzes potential solutions, including education, mentorship, and institutional support, to help anesthesiologists navigate this complex landscape more confidently and ethically. This review is intended to assist anesthesiologists with these various issues and is not intended to argue for or against transplant procedures.

## Review

Methods

This narrative review used a structured search strategy to identify relevant literature on anesthesiology and moral distress in organ procurement. Searches were performed across multiple databases, including Ovid MEDLINE, Embase, Web of Science, and Google Scholar. A combination of keywords and phrases was used to ensure comprehensive topic coverage.

The search terms included variations of "anesthesiology", "moral distress", "bioethics", "dead donor rule", "Uniform Determination of Death Act", "brain death", "circulatory death", and "normothermic regional perfusion". Specific terms focused on the ethical and psychological challenges in organ donation and the role of anesthesiologists.

A total of 77 articles were identified initially. After screening for relevance and applying inclusion criteria, which focused on ethical challenges, psychological impacts, or educational interventions related explicitly to anesthesiologists in organ donation, 18 articles were included in the final review (Appendix). Studies addressing other medical specialties or needing more substantial analysis of the targeted themes were excluded. Of note, if the search terms associated with anesthesiologists were excluded, the results yielded 11,371 articles.

Review

Dead Donor Rule

The DDR is central to maintaining ethical integrity in organ transplantation, mandating that organ retrieval must occur only after death has been definitively declared. The “dead donor rule,” which followed, is a philosophical synthesis of the UDDA and homicide law and establishes that no organ may be procured from anyone who is not pronounced dead by one or the other of these criteria [7]. For anesthesiologists, DDR serves as a professional and ethical boundary, yet it introduces considerable challenges for participation in NRP procedures. Balogh et al. described the operational tension anesthesiologists face in balancing their role in preserving organ viability with the imperative to uphold DDR. This dual obligation burdens the anesthesiologists in both donation after brain death and DCD. In brain death scenarios, discrepancies in definitions of brain death create uncertainties that place anesthesiologists involved in organ procurement in ethical dilemmas [8].

Glazier and Capron and Van Norman highlighted how the UDDA adopted by all U.S. states provides a legal framework for brain death but fails to account for variations in its interpretation, leading to ethical and legal challenges [1,9]. These challenges intensify when families contest the concept of brain death, often due to cultural or religious objections. Iaroshetskii et al. described how the lack of global consensus on death determination exacerbates these issues, especially for anesthesiologists working in multicultural settings [10]. The situational ambiguity inherent in DDR’s application not only complicates the ethical framework but also increases the emotional burden for anesthesiologists involved in organ donation.

In DCD cases, DDR requires that circulation and respiration cease irreversibly before organ procurement. Zimmermann et al. noted the temporal constraints associated with ensuring both the permanence of circulatory cessation and the preservation of transplantable organs [11]. This dichotomy creates ethical tension for anesthesiologists tasked with ensuring compliance while managing clinical processes to optimize organ viability.

The brain death controversy has been embedded with a very subtle move away from “irreversible” to “permanent” loss of brain function, introduced by Bernat, which, if appropriate, moves us away from a biological criterion for death to a “decisional” criterion [12]. This, in turn, might be considered to be the gateway to moral distress. NRP is grounded in this subtle move.

Normothermic Regional Perfusion and Ethical Ambiguity

NRP is an innovative technique that restores circulation to selected organs after death is declared, offering improved outcomes in organ preservation and transplantation. Currently, 49 organ procurement organizations facilitate NRP procedures in the United States. In this procedure, the donor is transported to the operating room, where ventilation is discontinued, and the heart stops. After waiting for a defined period of 5 to 30 minutes, extracorporeal membrane oxygenation (ECMO) is initiated, restarting circulation. Arteries are clamped to the head, and the heart is restarted to restore function. This differs from the traditional ex vivo or "heart-in-a-box" procedure, where the heart is preserved and maintained in cold storage outside the body [13].

However, the practice raises significant ethical concerns, particularly regarding its alignment with DDR. Katznelson et al. described how NRP challenges the finality of circulatory death by reintroducing circulation, which may appear to reverse the determination of death [14]. For anesthesiologists, this practice creates moral ambiguity, as it potentially conflicts with the public perception of death as an irreversible state.

Englbrecht et al. emphasized that the lack of standardized guidelines for NRP exacerbates these ethical concerns. In jurisdictions where NRP is accepted, anesthesiologists must navigate complex institutional protocols that may need more clarity on the role of partial circulation restoration [15]. Baumann et al. highlighted that inconsistencies in NRP implementation across regions further complicate anesthesiologists’ roles, often leaving them without sufficient ethical guidance [16]. This lack of uniformity places anesthesiologists at risk of moral distress, particularly when they feel that their professional duty could undermine the trust and ethical standards the public upholds them to within the organ donation process.

Zimmermann et al. described how NRP-related practices can lead to ethical dilemmas affecting donor families and clinicians. For anesthesiologists, reconciling their role in facilitating NRP with the moral imperatives of DDR requires robust ethical education and institutional support to ensure that their decisions align with professional standards and societal expectations [11].

Moral Distress and Moral Injury

Moral distress occurs when anesthesiologists cannot act according to their ethical beliefs, leading to severe professional and personal conflict. Mutlu and Utku identified a lack of standardized protocols and inadequate training as primary factors contributing to moral distress, particularly in scenarios involving brain death or circulatory death determinations [17]. This is further exacerbated when anesthesiologists must navigate culturally or religiously sensitive situations, as highlighted by Roudies et al., who emphasized the increased emotional burden these conflicts impose [18].

Moral distress may also emerge from the inherent tension between the ethical obligations to the deceased donor and the recipient, which are, to some extent, in conflict with one another. In certain circumstances, the anesthesiologist is required to perform interventions on the deceased donor that, while essential for facilitating the transplantation process, may be perceived as conflicting with the ethical considerations regarding the donor's dignity.

Moral distress, if left unaddressed, has the potential to evolve into moral injury. This progression occurs due to the cumulative nature of unresolved moral distress, often called the "crescendo effect" [19]. As individuals continue to experience situations that provoke moral distress, the accumulation of moral residue intensifies, leading to progressively higher levels of distress each time a morally troubling event occurs. Over time, this escalation can have profound psychological and emotional consequences, further exacerbating the impact of the initial distressing experience, ultimately ending in moral injury.

Moral injury refers to the intense cognitive and emotional reaction that can arise when events occur that breach an individual's moral or ethical beliefs more profoundly. Experiencing events that may be morally injurious can lead to intense feelings of shame and guilt, changes in thoughts and beliefs (e.g., "I am not good enough"), and unhealthy coping behaviors (such as substance abuse, social isolation, or self-harm). Zimmermann et al. highlighted that prolonged exposure to ethical uncertainties significantly contributes to burnout among anesthesiologists, leading to heightened emotional exhaustion and diminished professional fulfillment [11]. Englbrecht et al. further associated these outcomes with a lack of institutional resources to support anesthesiologists in addressing ethical conflicts [15]. Burnout affects individual anesthesiologists and impacts team dynamics and patient care, underscoring the importance of systemic interventions.

Institutional Support and Policy Needs

Structured institutional policies are critical in reducing moral distress and preventing burnout among anesthesiologists. Sanner et al. advocated for implementing clear guidelines and using ethics committees to provide support during ethically complex situations [20]. These committees enable collaborative decision-making, reducing the sense of isolation often associated with ethical dilemmas.

Iaroshetskii et al. emphasized the importance of regional adaptability in institutional policies, arguing that a one-size-fits-all approach is insufficient in addressing the diverse legal and cultural contexts anesthesiologists encounter [10]. Englbrecht et al. recommended proactive measures, such as ethics consultations and educational training programs, to empower anesthesiologists with the tools to navigate complex ethical scenarios confidently [15].

Addressing Knowledge Gaps

Addressing knowledge gaps is essential to equipping anesthesiologists with the skills to manage challenges effectively. Pence et al. advocated for integrating comprehensive ethics training into anesthesiology curricula, focusing on DDR, brain death criteria, and the ethical complexities of NRP [21]. This training may enhance their decision-making abilities while reducing the psychological burden of moral uncertainty.

Moguilevitch and Rufino emphasized the value of hands-on educational interventions, such as workshops and simulations, in preparing anesthesiologists for real-world ethical dilemmas [22]. These targeted programs allow anesthesiologists to explore complex scenarios in a controlled environment, fostering confidence and competence.

Ethics Curriculum and Ongoing Education

Ethics education should remain a cornerstone of professional development for anesthesiologists. Pence et al. advocated for integrating ethics modules into medical school and residency curricula, ensuring that clinicians are regularly exposed to evolving ethical standards throughout their careers [21]. Roudies et al. stressed the importance of culturally relevant training, especially for anesthesiologists in diverse clinical and regional contexts, to help them navigate unique challenges in transplantation practices [18].

Continuing medical education programs and interdisciplinary seminars provide essential opportunities for anesthesiologists to stay updated on advancements in ethical frameworks and organ procurement technologies. These initiatives are crucial for fostering ethical competence, reducing the risk of moral injury, and equipping practitioners with the tools to navigate complex scenarios confidently. Furthermore, engaging in mentorship programs and peer-to-peer support networks offers valuable opportunities for shared learning and collaboration.

Structured debriefs, pastoral care assistance, and access to psychological support services create safe spaces for anesthesiologists to reflect on challenging experiences and address the emotional toll of moral distress. Active involvement in developing health system policies, participation in ethics committees, and contribution to quality improvement and performance improvement projects allow anesthesiologists to influence systemic practices and foster ethical clarity. Additional efforts, such as education in transplantation protocols, building cognitive and communication skills, and identifying organizational and systemic factors contributing to moral distress, are integral to creating a supportive and ethically sound professional environment. By prioritizing these strategies, the field can ensure that anesthesiologists are well-prepared to uphold ethical standards and maintain professional integrity.

## Conclusions

Ethical dilemmas in organ procurement, particularly those surrounding DDR and NRP, pose significant challenges for anesthesiologists. These complexities often lead to moral distress as anesthesiologists navigate conflicting professional responsibilities, legal inconsistencies, and evolving medical practices. The psychological toll, including moral distress, moral injury, and burnout, underscores the necessity for robust systemic support. Addressing these challenges requires a comprehensive approach incorporating clear institutional protocols, targeted ethics education, and access to ethics consultation services.

As the prevalence of NRP increases, the need for anesthesiologists to receive increased education and support becomes more urgent. Standardized guidelines, alongside ongoing research into the ethical and psychological impacts of emerging organ procurement techniques, are critical to ensuring that anesthesiologists can maintain ethical integrity while navigating these complex scenarios. Given the concern of moral distress, a preventive approach rather than a reactive one may be productive. Developing an educational curriculum and encouraging discussion with peer support and mentors may normalize procurement procedures and help resolve individual conflicts. By prioritizing education, mentorship, and institutional frameworks, the field can empower anesthesiologists to fulfill their roles confidently, safeguarding professional well-being and public trust in organ donation.

The results of this review suggest that more research is needed to understand why anesthesiologists experience moral distress with organ procurement. Additionally, research is required to know how best to identify, prevent, and treat moral distress in this population. Specialty societies, healthcare institutions, and training programs should support these efforts by routinely providing opportunities for education, mentorship, and support services.

## Appendices

**Table 1:** Summary table NRP, normothermic regional perfusion; NRP-cDCD, normothermic regional perfusion with controlled heart donation after circulatory determination of death; PMOD, postmortem organ donation; DCD, donation after circulatory death

Author	Year	Article Type	Journal	Plain Language Summary
Glazier et al. [1]	2022	Editorial	Am J Transplant	The practice of NRP raises potential conflicts with the Uniform Determination of Death Act.
Truog et al. [3]	2023	Viewpoint	JAMA	NRP involves the use of extracorporeal membrane oxygenation to restore perfusion to internal organs in situ before they are removed from a deceased donor.
Parent et al. [4]	2020	Personal Viewpoint	Am J Transplant	NRP-cDCD presents ethical and legal concerns.
American College of Physicians (ACP) [5]	2021	Position Statement	N/A	ACP recommends pausing the use of NRP-cDCD due to the lack of ethical and legal standards of the practice.
American Society of Transplantation [6]	2022	Website Page	N/A	The American Society of Transplantation shares their statement regarding NRP.
Balogh et al. [8]	2022	Full Text Review Article	Transplantation Reports	Anesthesiologists are vital to the organ donation process and should continue intensive care protocols perioperatively, despite limited guidance on analgesia and sedation. Familiarity with institutional policies for brain and cardiac death donations, along with resources from professional associations, supports their role.
Van Norman [9]	1999	Case Report	Anesthesiology	As these cases show, anesthesiologists play a crucial role in ensuring living patients are not sacrificed for organ donation. They must be thoroughly familiar with the criteria for declaring death when involved in organ retrieval.
Iaroshetskiĭ [10]	2010	Viewpoint	Anesteziol Reanimatol	The organizational, legal, and ethical issues of organ donation for transplantation are examined from the perspective of an anesthesiologist-resuscitation specialist, highlighting the parallels between managing a brain-dead donor and treating a patient with multiple organ dysfunction.
Zimmermann et al. [11]	2019	Full Text Review Article	Am J Transplant	The findings from a qualitative study using semi-structured interviews and focus groups indicate that while professional stakeholders consider definitions of death and clear rules around organ donation are essential, donor families place less importance on these concepts when judging the acceptability of donation.
Sellers et al. [13]	2024	Original Investigation	JAMA	Findings from survey study of US organ procurement organizations indicate that better standardization of NRP policies, protocols, and practices is both needed and desired to optimize organ donation and transplantation outcomes from donors after circulatory determination of death.
Katznelson et al.[14]	2018	Special Article	Anaesthesiol Intensive Therapy	This paper re-examines the anesthesiologist’s role in administering analgesics during organ procurement, tracing the historical and ethical foundations of the practice, including brain death and the 'dead donor rule.' It argues for a potential re-framing of these ethics to balance donor protection with public trust in medicine.
Englbrecht et al.[15]	2022	Review	Anaesthesist	This review guides anesthetists in organ-protective perioperative therapy, summarizing pathophysiological changes in brain-dead patients. It examines evidence, guidelines, and ethical considerations related to treatment strategies and anesthesia during organ retrieval.
Baumann et al.[16]	2017	Editorial	Eur J Anaesthesiol	The text explores the ethical and practical implications of presumed consent (opt-out) systems for PMOD, which are increasingly adopted in Europe to address organ shortages. While such systems may improve donation rates and align with utilitarian public health goals, they raise concerns about individual autonomy, possible manipulation, and the emotional impact on relatives who may feel excluded or pressured. The author argues that more ethical and compassionate PMOD practices should involve relatives in the decision-making process, supported by trained medical staff, to honor the potential donor’s values and reduce family distress.
Mutlu et al.[17]	2019	Full Text Article	Transplant Proc	Data from questionnaires filled out by professionals in anesthesiology and reanimation or/and intensive care identify a lack of standardized protocols and inadequate training as primary factors contributing to moral distress, particularly in scenarios involving brain death or circulatory death determinations.
Roudies et al. [18]	2013	Full Text Article	Transplant International	Moral distress is further exacerbated when anesthesiologists must navigate culturally or religiously sensitive situations.
Sanner at al. [20]	2006	Original Article	Intensive Care Medicine	A survey sent to anesthetists and neurosurgeons in intensive care units identified obstacles in organ donation—neutral donation requests, ethical concerns, diagnostic variability, and limited ICU beds—require targeted efforts to improve organ donation rates. Monitoring these factors can serve as quality control in evaluating hospital donation practices.
Pence et al. [21]	2024	Research Article	Camb Q Healthc Ethics	Anesthesiology training programs lack a standardized ethics curriculum despite the need to prepare trainees for medical and ethical competence and board certification. To address this, a 2021 survey of biomedical ethics experts identified and ranked the top ten essential ethics topics for anesthesiology education, including consent capacity, surrogate decision-making, DNR status, patient autonomy, end-of-life care, medical error disclosure, brain death criteria, and impaired anesthesiologists.
Moguilevitch et al. [22]	2023	Review Article	Transplantation	This paper examines the challenges and supports affecting physician anesthesiologists' involvement in DCD at academic medical centers. DCD is vital for expanding the organ donor pool but involves complex roles for anesthesiologists in both end-of-life care and organ procurement.
